# Decreased exercise capacity in ‘asymptomatic’ patients late after relief of severe pulmonary stenosis and moderate restenosis: evidence for diastolic dysfunction

**DOI:** 10.1186/1532-429X-14-S1-O80

**Published:** 2012-02-01

**Authors:** Soha Romeih, Nico A Blom, Mart N van der Plas, Anje M Spijkerboer, Barbara J Mulder, Maarten Groenink

**Affiliations:** 1 Cardiology, Academic Medical Center, Amsterdam, Netherlands; 2 Radiology, Academic Medical Center, Amsterdam, Netherlands; 3 Pediatric Cardiology, Academic Medical Center, Amsterdam, Netherlands; 4 Pulmonology, Academic Medical Center, Amsterdam, Netherlands

## Summary

We concluded that close and regular follow up of asymptomatic patients with a history of previous intervention for PS and residual moderate PS by stress testing is recommended. This might reveal cardiac functional deterioration, which could be an indicator for the earlier intervention in this group of patients to avoid permanent cardiac dysfunction.

## Background

Little is known about the management of moderate pulmonary restenosis after relief for severe pulmonary valve stenosis (PS).We evaluated cardiac response to physical and pharmacological stress late after relief of severe PS in these patients.

## Methods

Twenty asymptomatic patients with moderate PS were divided into 2 groups: Group I (late after relief of severe PS, n = 9), and Group II (no previous intervention, n = 11). All patients underwent an exercise test, dobutamine stress (DS) MRI, and delayed contrast enhanced MRI. The response to physical and pharmacological stress was compared between both groups.

## Results

**Figure 1:**
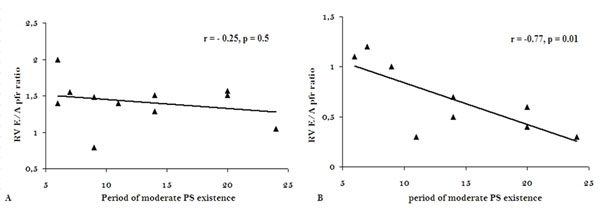
Correlation between RV E/A peak flow rate ratio and the period of moderate PS existence (A) Group I (B) Group II.

Group I showed impaired exercise capacity compared to Group II (VO2max = 72.8 ± 3.5 vs. 103 ± 16%, p = 0.01). At rest, both groups showed normal systolic function. Majority of Group I patients have impaired RV diastolic function (82% vs. 33%, P = 0.004). During DS-MRI, right ventricular stroke volume (RV-SV) increased in Group II but not in Group I (+13 ± 8 ml, - 5 ± 8 ml, p < 0.001). In Group I, RV peak flow rate ratio correlated positively with Δ RV-SV (r = 0.58, p = 0.01), and negatively with the period of moderate PS existence (r = -0.77, p = 0.01- Figure 1). VO2max and O2-pulse were correlated with RV-SV response to DS-MRI.

## Conclusions

Impaired exercise capacity in patients with moderate pulmonary restenosis after relief of severe PS is caused by inability to increase RV-SV. This is probably caused by disturbed RV filling properties, which worsen in time.

## Funding

Financial disclosure: none.

